# A New Approach to Quantifying Bioaccumulation of Elements in Biological Processes

**DOI:** 10.3390/biology10040345

**Published:** 2021-04-20

**Authors:** Kinga Proc, Piotr Bulak, Monika Kaczor, Andrzej Bieganowski

**Affiliations:** Institute of Agrophysics, Polish Academy of Sciences, Doświadczalna 4, 20-290 Lublin, Poland; k.proc@ipan.lublin.pl (K.P.); m.kaczor@ipan.lublin.pl (M.K.); a.bieganowski@ipan.lublin.pl (A.B.)

**Keywords:** bioaccumulation factor, bioaccumulation index, entomoremediation, insects

## Abstract

**Simple Summary:**

The bioaccumulation of elements (e.g., heavy metals) in living organisms (e.g., animals) is vitally important from at least two points of view: the growth and development of the organisms themselves and remediation of the polluted environment. So far, bioaccumulation has been characterized by the bioaccumulation factor (BAF), which is the ratio between the concentration of elements in the organism to the concentration in the matrix (water, soil, etc.). This factor is a good measure of bioaccumulation in ecosystems in which an organism lives from the beginning of their lives to the moment of investigation. However, especially in laboratory experiments, when organisms at a given stage of development are introduced to the system and contain some non-zero concentration of an element, the BAF can lead to misinterpretation. Therefore, we propose a new measure called the bioaccumulation index (BAI), which is the relative increase in the concentration of a given element in the organism to its initial concentration after the experiment. We proved, on the basis of data published by other authors, that the BAI was much more valid for the interpretation of bioaccumulation in these cases.

**Abstract:**

Bioaccumulation, expressed as the bioaccumulation factor (BAF), is a phenomenon widely investigated in the natural environment and at laboratory scale. However, the BAF is more suitable for ecological studies, while in small-scale experiments it has limitations, which are discussed in this article. We propose a new indicator, the bioaccumulation index (BAI). The BAI takes into account the initial load of test elements, which are added to the experimental system together with the biomass of the organism. This offers the opportunity to explore the phenomena related to the bioaccumulation and, contrary to the BAF, can also reveal the dilution of element concentration in the organism. The BAF can overestimate bioaccumulation, and in an extremal situation, when the dilution of element concentration during organism growth occurs, the BAF may produce completely opposite results to the BAI. In one of the examples presented in this work (Tschirner and Simon, 2015), the concentration of phosphorous in fly larvae was lower after the experiment than in the younger larvae before the experiment. Because the phosphorous concentration in the feed was low, the BAF indicated a high bioaccumulation of this element (BAF = 14.85). In contrast, the BAI showed element dilution, which is a more realistic situation (BAI = −0.32). By taking more data into account, the BAI seems to be more valid in determining bioaccumulation, especially in the context of entomoremediation research.

## 1. Introduction

The term “bioaccumulation”, usually expressed as the bioaccumulation factor (BAF), is used to describe the metabolism-mediated active transport of xenobiotics (such as metals) from the environment to a living organism, which are then accumulated intracellularly. These substances can be, for example, pesticides [1], heavy metals [2] or other elements [3], mycotoxins [4], and persistent organic pollutants [5]. The spectrum of living organisms is also wide, as BAFs are used to describe bioaccumulation in animals such as fishes [6], insects [2], snails [7], and cattle [8]; in plant roots, leaves, and stems (e.g., halophytes) [9]; as well as in microorganisms such as bacteria [10] and microalgae [11].

The most commonly used formula for calculating the BAF is as follows [12]:

(1) $$\mathrm{BAF}=\frac{C_{\mathrm{substance}\;\mathrm{in}\;\mathrm{the}\;\mathrm{organism}}}{C_{\mathrm{substance}\;\mathrm{in}\;\mathrm{the}\;\mathrm{matrix}}}$$

where C is the concentration and can be expressed in mass unit per mass or volume unit.

A review of the literature leads to the conclusion that the first works describing bioaccumulation were devoted to ecosystem monitoring [12,13,14,15], and this remains an area of interest for researchers [16,17]. However, as noted by [18], bioaccumulation assays are also conducted under laboratory conditions.

There are many examples of such investigations [19,20,21,22], and experiments with entomoremediation are an emerging area. This new subtype of bioremediation utilizes specialized insects and associated microorganisms to extract, sequester, and/or detoxify pollutants from soil, sediments, and organic biomass [23,24]. Several different insects, such as ants, beetles, termites, as well as Collembolans, were originally proposed as entomoremediators [23].

Similarly to phytoextraction, entomoextraction can be defined as the sequestration and extraction of heavy metals (or elements in general) into insect exoskeletons or other easily obtained insect body parts [25,26]. Thus, the most interesting in this regard are insects which bioaccumulate heavy metals and other elements. *Hermetia illucens* (Diptera: Stratiomyidae) is the best example of an insect recently used in experiments in entomoremediation and entomoextraction due to its ability to bioaccumulate different elements (e.g., Ca, Cd, Mn) [2,24,26,27,28].

The BAF, as the quantity describing the accumulation of investigated substances, was developed for large ecosystems but then directly transferred to small-scale experiments. The question arises: is this transfer valid? In other words, does the use of formulas valid for large scales remain appropriate for small mass/volume laboratory research?

The aim of this work is to critically discuss the interpretation of the use of the BAF in small-scale laboratory experiments and to propose a new indicator that is more valid in describing bioaccumulation phenomena of various chemical elements, especially in the context of entomoremediation research.

## 2. Materials and Methods

### 2.1. Data Selection

We calculated the BAF as well as the new bioaccumulation indicator based on published data from the experiment in [27]. The authors carried out an experiment in which *Hermetia illucens* (black soldier fly, BSF) larvae were bred on three different substrates (i.e., experimental matrices). These substrates were (i) a mixture of middlings, referred to as a balanced substrate for the control group; (ii) dried distillers’ grains rich in proteins; and (iii) dried sugar beet pulp rich in fiber (a full characteristic of the substrates is in the cited work). Before the experiment, the *H. illucens* larvae were bred in commercial feed for turkeys and chickens.

These data are excellent for showing differences between the BAF and the proposed new bioaccumulation indicator because they relate to three different substrates tested in one experimental setup and they present concentrations of different elements. However, we chose three elements: phosphorous, manganese, and cadmium. Because the aim of this work was to show a new approach to measuring bioaccumulation, we selected those elements that clearly illustrate the main difference between both approaches, the BAF and the BAI. From this point of view, it was not important whether these elements were nutritive or toxic, and their role in larval metabolism was irrelevant.

### 2.2. Bioaccumulation Index (BAI)

A new measure for bioaccumulation is proposed, given by Equation (2),

(2) $$\mathrm{BAI}=\frac{C_{\mathrm{in}\;\mathrm{organism}\;\mathrm{after}\;\mathrm{experiment}}-C_{\mathrm{in}\;\mathrm{organism}\;\mathrm{before}\;\mathrm{experiment}}}{C_{\mathrm{in}\;\mathrm{organism}\;\mathrm{before}\;\mathrm{experiment}}}$$

where BAI is the bioaccumulation index; C_in organism after experiment_ is the concentration of a given element in DW of organism biomass after the experiment (mass unit per mass unit); and C_in organism before experiment_ is the concentration of a given element in DW of organism biomass before the experiment (mass unit per mass unit).

It can be seen from Equation (2) that from a mathematical point of view, the BAI represents the relative increase in the concentration of a given element to its initial concentration.

## 3. Results

For all cited cases (except one), the BAFs were greater than 1, which indicates that bioaccumulation occurred (Table 1). Only for P in the protein substrate was the BAF less than 1, indicating that there was no bioaccumulation in this case. The BAI took negative values in the case of P in all substrate variants and for Mn in the protein substrate. The BAI was higher than 0 in the case of Cd in all substrates and for Mn in the control and fiber substrates.

The highest BAF was obtained for P in the fiber substrate, and reached 14.85. The amount of P in the fiber substrate was low (0.89 g·kg^−1^), but the input of this element in the biomass of young larvae was high (19.51 g·kg^−1^). However, the final amount of P in the larvae at the end of the experiment was lower (13.22 g·kg^−1^).

## 4. Discussion

### 4.1. Meaning and Limitations of the BAF

As mentioned previously, the BAF is a measure dedicated to characterizing the bioaccumulation of different substances in living organisms in the natural environment (hereafter “natural matrix”) and directly transferred to laboratory-scale experiments. When an organism is born into a given natural matrix, e.g., a lake, and during its lifespan the lake is the only place it lives, bioaccumulation takes place from birth to death or to the moment when the organism is sampled for measurement. In these situations, the concentration of a given element in the biomass of this organism can be related solely to the concentration in the natural matrix, according to Equation (1), and in this context, the BAF is absolutely valid.

However, the question arises as to how this measure can characterize experiments done on a small scale under laboratory conditions, in which the younger form of an organism is taken from the stock colony or obtained from external sources and then placed on a completely different feed used in the experiment. Along with the addition of these young organisms, the elements accumulated thus far in their biomass are being added to the system.

This situation occurs much less frequently in the ecological investigations from which Equation (1) was derived. Consequently, the use of the BAF in laboratory experiments can lead to mistakes in interpretation. Depending on the mutual ratios between the initial element concentration in the biomass of an organism and the experimental matrix, the concentration of a given element in the biomass of the organism can increase, decrease, or stay unchanged. Cases where there is a decrease in element concentration cannot be recognized using the BAF. Moreover, there are situations when the use of the BAF in small-scale laboratory experiments can lead to incorrect interpretations of real phenomena, which will be discussed later.

### 4.2. The Concept and Biological Meanings of the BAF and the BAI—Different Approaches to Bioaccumulation

The BAF can obtain three threshold values but cannot have negative values. When the concentration of an element in an organism is higher than in the natural matrix, the BAF takes values greater than 1, which means that bioaccumulation occurred (Table 2). If the relationship between the concentrations is reversed (i.e., BAF < 1), bioaccumulation did not occur. A special case is where BAF = 1. In these cases, the concentration of an element in the organism corresponds to the concentration in its environment. This situation can be referred to as bioindication, and organisms that show this tendency can be called bioindicators [29,30]. In some organisms, this dependence can exist in a wide range of element concentrations and can be used in environmental monitoring. Examples of such organisms, used for the biomonitoring of radionuclides, toxic heavy metals, and pesticides, are freshwater mussels and phytoplankton, bees, earthworms, lichens [30], fungi [31], and small soil invertebrates [32].

The BAI can have two other threshold values that cannot be expressed by the BAF: it can be equal to 0 and it can be negative. However, the same value obtained for both indicators will mean different things in some cases (Table 2). For example, BAI = 1 does not indicate bioindication, as the BAI does not refer to the content of elements in the environment. In such cases, it is only possible to state that bioaccumulation took place and that the concentration of a given element in the biomass of the organism after the experiment was exactly twice its initial concentration. A similar example can be seen in the case of Mn in the fiber matrix, where the BAI was 0.92 and the BAF was 6.00 (Table 1). Both measures (BAF and BAI) indicated bioaccumulation; the BAF indicated that the concentration of Mn in the larvae was six times higher than in the matrix, but the BAI indicates that the Mn concentration in the larvae after the experiment was nearly double the concentration in the larvae before the experiment (Table 1).

An excellent example of the power of the BAI is illustrated in every case where this measure took negative values (Table 1). These were cases of element dilution. The specific reason for an element “leaching” from an organism may not necessarily be active removal of the element during the organism’s growth. The element may have been very minimally available from the substrate, therefore it may be a case of growth dilution [32] (i.e., a relative reduction in the concentration of an element in the body of an organism due to an increase in the body mass or volume). This is the essence of the BAI’s improvement over the classical BAF.

An interpretation made using the BAF in Table 1 could lead to the conclusion that *H. illucens* is a bioindicator for P in the control and protein substrates, as the calculated values of BAF were close to 1. However, analysis of the BAI values reveals the possibility of another interpretation: in both cases, P was transferred from larvae to the matrix (dilution). Phosphorus in the fiber substrate seems to be the best example to demonstrate the confusion that can arise from using the BAF. The high BAF value for P indicated bioaccumulation; however, the BAI showed a negative value, which pointed to P dilution. The situation is clearly seen when studying the change in P concentration in *H. illucens* larvae, which was higher in young larvae than in larvae after the experiment. The conclusion is that during the course of the experiment, the concentration of P decreased in the larvae’s bodies. The BAF value was especially high because of the small amount of P in the substrate and the high concentration of P in the young larvae.

The results of the BAF calculated for Cd (Table 1) indicated bioaccumulation. In all cases, bioaccumulation was also confirmed by the BAI.

The question may arise: which measure of bioaccumulation (BAF or BAI) is better? The simple answer is that it is not possible to show which is better, as they each characterize bioaccumulation from a different point of view and in different systems. However, without additional information about initial and final element concentrations in the biomass of an organism, it is not possible to calculate the BAI and state whether the BAF overestimates bioaccumulation. Therefore, the BAI is a much more appropriate measure when initial and final concentrations of a given element are known.

The term “bioaccumulation” in the majority of papers is strictly related to Equation (1). Many recent publications have followed this approach [33,34]. Therefore, some authors have treated the equation as the definition of bioaccumulation. However, it should be remembered that this is a narrow definition, valid specifically for studying the natural environment.

A wider definition of bioaccumulation was also proposed: bioaccumulation is any case where the concentration of an element/substance increased in an organism during its growth [35,36]. This approach opens up the possibility of using measures of bioaccumulation other than the BAF—for instance, the BAI (Equation (2)).

As mentioned previously, the BAF was developed for environmental investigations. This fact is hugely important to properly understand the physical meaning of Equation (1). If bioaccumulation takes place in an ocean, sea, or river, it can be assumed (without any measurable error) that the uptake of elements does not decrease their concentration in the natural matrix (i.e., in the ocean, sea, or river). This means that the denominator in Equation (1) is constant before and after the experiment and that the BAF calculated according to Equation (1) indicates the concentration of a bioaccumulated substance in relation to the constant concentration in the environment in which the organism lives.

The situation may be completely different when the experiment is carried out on a laboratory scale. In addition to the previously discussed problem of introducing the mass of the elements together with the organism’s biomass into the experimental system from the initial colony, the problem of feed limitation can also arise. In extreme situations, it is possible for all nutrients to be consumed completely by the investigated organism. This problem has been confirmed by studies in which there was a need to determine the optimal dose of food [2,36]. If this is correct, the denominator in Equation (1) will be dependent on a combination of the initial concentration of elements and the uptaken (eaten) amount of the investigated substance (i.e., indirectly on the amount and activity of the tested organism). It is obvious that bioaccumulation in a system with an abundance of feed will be different from that in a system with a deficit in feed. This may lead to difficulties in comparing the results obtained in different experiments/laboratories, even for the same investigated organisms, and hence the need to precisely define what mass of the substrate (mg or g) was given per test organism.

### 4.3. The Applicability of the BAI for Other Organisms

It is not easy to find in the literature a set of data that would allow calculation of both BAF and BAI indicators; this is due to the common practice of not testing the content of elements in the organism before the experiment. However, we found data to calculate the BAI (and to make a comparison with the BAF) for two completely different animals: *Paracyclopina nana* and *Helix aspersa*, which are planktonic copepods living in water and the common garden snail, respectively. The results are presented in Table 3. The criteria for elements and variant selection were the same as for Table 1.

As can be seen regarding Cd in *P. nana*, the BAF was below 1 and showed no bioaccumulation [37]. In contrast, the BAI indicated bioaccumulation, which can be confirmed by comparing the initial and final concentrations of the element in the organism. For an opposite result, in the case of Cu, the BAF showed very high bioaccumulation, while the BAI pointed to the dilution of this element (the final *P. nana* concentration was lower than the initial). As in the case of P from [27] and Table 1, this example shows a case where the interpretation of the values of both the BAF and BAI indicates completely different phenomena. For *H. aspersa* [38] in all cases, the interpretation of both indicators showed the same conclusion concerning bioaccumulation. As can be seen, the BAI can also be successfully applied to organisms other than *H. illucens*.

### 4.4. Limitations in Using the BAI

There are some disadvantages to the use of the BAI as a measure of bioaccumulation. The BAI has limited use when the biomass of an organism is divided into two fluxes of the biomasses. A very good example is *H. illucens*, a holometabolic insect that undergoes complete metamorphosis. The life of these insects consists of four developmental stages: egg, larvae, pupae, and adult individual (imago). After the pupae stage, the biomass is divided into the adult insect and the puparia (or pupal exuviae). Puparia are especially important from an entomoextraction point of view, as they may contain high amounts of bioaccumulated elements [2,24]. As the BAF is commonly calculated for all developmental stages, it is more difficult in the case of the BAI for puparia and the adult insect. The question arises: to which developmental stage should the BAI refer in the denominator of Equation (2)? Should the initial concentration in younger larvae be subtracted (Equation (2)) from the concentration in the puparia or imago (as stage “after”)? None of the answers seems applicable.

When the amount of the substance in the experimental matrix is limited and the experiment lasts longer or the number of organisms is high, bioaccumulation can be limited because of the lack of substrate. The value of the BAI (and that of the BAF) can be underestimated in these situations. Therefore, it is very important to report the initial and final concentrations of an element in the matrix in order to enable the appropriate interpretation of the results as well as the mass of the substrate per tested organism.

## 5. Conclusions

The bioaccumulation factor (BAF) is valid in environmental investigations. The concentration of a bioaccumulated element can be treated as constant before and after the experiment. The BAF does not provide information about the dilution of elements, but does make it possible to draw conclusions about bioindication.

The use of the bioaccumulation index (BAI) proposed in this work, which can be defined as the relative change in the concentration of a given element in the biomass of investigated organisms, makes it possible to better characterize the phenomenon of bioaccumulation in small-scale laboratory experiments, compared with the BAF. This is because the BAI takes into account the load of elements delivered into the experimental system along with the tested organisms. In laboratory-scale experiments, commonly used methodology involves the use of organisms that have been previously fed other feed and grown in conditions other than those ultimately present in the planned experiment. The BAI can also indicate cases of the dilution of elements in organisms. Nonetheless, the major disadvantage of the BAI is that it does not allow for the easy calculation of bioaccumulation when an organism’s biomass is divided into two fluxes of biomasses, as is often the case with insects.

## Figures and Tables

**Table 1:** The concentrations of selected elements in substrates used as feed for *Hermetia illucens* larvae and concentrations of these elements in the young larvae before (initial) and after (final) the experiment, according to publication [27] (gray cells). BAF and BAI values were calculated according to Equations (1) and (2), respectively.

	Element Concentration in Substrates for *H. illucens* Larvae			Element Concentration in the Larvae				BAF			BAI		
Element/Variant	Control	Protein Rich	Fiber Rich	Young (Initial)	Control (Final)	Protein Rich (Final)	Fiber Rich (Final)	Control	Protein Rich	Fiber Rich	Control	Protein Rich	Fiber Rich
P (g·kg^−1^)	7.74	9.55	0.89	19.51	8.91	8.71	13.22	1.15	0.91	14.85	−0.54	−0.55	−0.32
Mn (g·kg^−1^)	0.26	0.06	0.08	0.25	0.73	0.19	0.48	2.81	3.17	6.00	1.92	−0.24	0.92
Cd (mg·kg^−1^)	0.09	0.08	0.23	0.36	0.47	0.60	2.24	5.22	7.50	9.74	0.31	0.67	5.22

**Table 2:** The difference in the concept and the interpretation of different threshold values of both bioaccumulation measures.

		BAF	BAI
Describes the Ratio of the Final Concentration in the Biomass of Larvae in Relation to…		…the Concentration in the Matrix (e.g., in Sea, Lake, River).	…the Initial Concentration of a Given Element in the Biomass of the Younger Organism.
Threshold Values	BAF or BAI > 1	Bioaccumulation. The concentration of a given element in the biomass of an organism was higher than in the matrix.	Bioaccumulation. The concentration of a given element in the biomass of an organism after the experiment was higher than in the biomass of the same organism before the experiment. The relative concentration of the given element increased during the experiment.
	BAF or BAI = 1	Bioindication. The concentration of a given element in the biomass of an organism was equal to the concentration in the matrix, and such organisms can be treated as bioindicators of a given element in such environments.	Bioaccumulation. The final concentration of a given element in the biomass of an organism (after the experiment) was exactly equal to twice the initial concentration. There can be no question about bioindication, because the BAI does not refer to a given element concentration in the matrix.
	0 < BAF or BAI < 1	No bioaccumulation. The concentration of a given element in the biomass of an organism was lower than in the matrix.	Bioaccumulation. However, the extent of bioaccumulation was lower; the final concentration of a given element in the biomass of the organism was greater than the initial concentration but less than twice the initial concentration.
	BAF or BAI = 0	N/A	No bioaccumulation occurred, as the final concentration of a given element in the biomass of the organism was equal to the initial concentration.
	BAF or BAI < 0	N/A	The phenomenon of the “dilution” of an element occurred during the experiment. A given element was transferred from the biomass of the organism to the matrix; the final concentration in the organism’s biomass was lower than the initial value.

**Table 3:** Examples of the applicability of the BAI to organisms other than *Hermetia illucens*.

Organism	Element	Concentration in the Matrix (µg·g^−1^)	Initial Concentration in the Organism (µg·g^−1^)	Variant Names According to Cited Reference	Final Concentration in the Organism (µg·g^−1^)	BAF	BAF Interpretation	BAI	BAI Interpretation	Reference
*P. nana*	Cd	0.26	0.02	Cfood	0.16	0.62	–	7.00	+	[37]
				Cnofood	0.21	0.81	–	9.50	+	
	Cu	2.40	226.90	Mixfood	98.7	41.13	+	−0.57	–	
				Mixnofood	149.5	62.29	+	−0.34	–	
*H. aspersa*	Cd	0.12	0.16	Control	0.07	0.58	–	−0.56	–	[38]
		13.00	24.31	S10	38.11	2.93	+	0.57	+	
		54.76	99.67	S50	174.61	3.19	+	0.75	+	
		83.20	237.91	S100	282.33	3.39	+	0.19	+	

+ bioaccumulation occurred; – no bioaccumulation.

## Data Availability

Not applicable.
